# High efficacy of lopinavir/r-based second-line antiretroviral treatment after 24 months of follow up at ESTHER/Calmette Hospital in Phnom Penh, Cambodia

**DOI:** 10.1186/1758-2652-14-14

**Published:** 2011-03-26

**Authors:** Laurent Ferradini, Vara Ouk, Olivier Segeral, Janin Nouhin, Anne Dulioust, Chanroeurn Hak, Isabelle Fournier, Nathalie Lerolle, Sopheak Ngin, Chhi Vun Mean, Jean-François Delfraissy, Eric Nerrienet

**Affiliations:** 1 Family Health International, Phnom Penh, Cambodia; 2 ESTHER/Calmette Hospital, Phnom Penh, Cambodia; 3 Clinical Immunology Department, Bicêtre Hospital, Kremlin Bicêtre, France; 4 Laboratoire VIH/Hépatites, Institut Pasteur du Cambodge, Phnom Penh, Cambodia; 5 ANRS, Phnom Penh, Cambodia; 6 National Center for HIV/AIDS, Dermatology and STD, Ministry of Health, Cambodia; 7 ANRS, Paris, France

## Abstract

**Background:**

The number of patients on second-line highly active antiretroviral therapy (HAART) regimens is increasing in resource-limited settings. We describe the outcomes after 24 months for patients on LPV/r-based second-line regimens followed up by the ESTHER programme in Phnom Penh, Cambodia.

**Methods:**

Seventy patients who initiated second-line HAART regimens more than 24 months earlier were included, and immuno-virological data analyzed. HIV RNA viral load was determined by real-time RT-PCR. HIV-1 drug resistance was interpreted according to the ANRS algorithm.

**Results:**

Of the 70 patients, two were lost to follow up, three died and 65 (92.8%) remained on second-line treatment after 24 months of follow up (median duration of treatment: 27.4 months). At switch to second-line, the median CD4 T cell count was 106 cells/mm^3 ^and the median viral load was 4.7 Log_10_. Second-line regimens prescribed were ddI/3TC/LPV_/r _(65.7%), ddI/TDF/LPV_/r _(10.0%), ddI/AZT/LPV_/r _(8.6%) and TDF/3TC/LPV_/r _(7.1%). The median CD4 T cell gain was +258 cells/mm^3 ^at 24 months (n = 63). After 24 months of follow up, 92.3% (60/65) of the patients presented undetectable viral loads, giving an overall treatment success rate of 85.7% (CI: 75.6- 92.0) in intent-to-treat analysis.

**Conclusions:**

These data suggest that a LPV_/r_-based second-line regimen is associated with a high rate of virological suppression and immune reconstitution after 24 months of follow up in Cambodia.

## Background

Highly active antiretroviral therapy (HAART) programmes have proven the feasibility and efficacy of first-line HAART regimens in resource-limited settings, similar to those reported in developed countries [1-10]. As initially emphasized stimulated by non-governmental organizations and the World Health Organization's (WHO's) "3 by 5" initiative, increasing numbers of patients are now initiating first-line HAART regimen in African and Asian cohorts [11-14]. At the same time, the duration of follow up of patients on HAART is increasing and treatment failures are becoming more common, with an increasing number of patients having to start second-line HAART regimens in such settings [15,16].

Previous WHO recommendations for second-line regimens proposed the choice of antiretroviral (ARV) drug combinations, including two distinct nucleoside reverse transcriptase inhibitors (NRTI), as didanosine (ddI), abacavir (ABC), tenofovir (TDF) or lamivudine (3TC), and one ritonavir-boosted protease inhibitor (PI/r) [17]. More recently, WHO Rapid Advice Guidelines recommend the use of TDF and 3TC or emtricitabine (FTC), or zidovudine (AZT) and 3TC as the NRTI backbone, together with one PI/r (LPV/r or ATV/r) [18].

However, because of the limits imposed by first-line regimen options (limited number of available drugs, including a thymidine analog) and because some second-line drugs are either poorly available or prohibitively expensive, the choice of second-line strategies becomes an important and difficult issue for both the patients and national programmes [19]. Few data on the feasibility and efficacy of such second-line regimens in resource-limited settings have been published so far [20-24]. Such information would be useful for national programmes in choosing the most appropriate and affordable second-line combinations.

In Cambodia, the latest estimated HIV prevalence of 0.9% at the end of 2006 among adults (15-49 years) remains one of the highest rates in south-east Asia [25,26]; estimates are that almost 67,200 people are infected with HIV. HAART was introduced in the country in 2001, and after a period of active scaling up, the National Center for HIV/AIDS, Dermatology and STD (NCHADS) recently reported that 33,287 patients were on HAART by the end of March 2009 [27]. Treatment failures in Cambodia are detected mostly by using immunological criteria since HIV viral load (VL) is not already used in routines for virological follow up.

The number of patients already on PI-based regimens was estimated in March 2009 to be around 1145 adults, which represents 3.9% of adult patients on HAART in the country. The present study reports on the outcomes of HIV adults on lopinavir/ritonavir (LPV/r)-based second-line HAART regimens for more than 24 months, followed up by the ESTHER programme at the Calmette Hospital in Phnom Penh, Cambodia.

## Methods

### Setting

All patients evaluated in this study were part of the ESTHER cohort, followed up at the Calmette Hospital. The French ESTHER programme was implemented in the Calmette Hospital in February 2003 in collaboration with the Cambodian Ministry of Health. HAART initiation began in July 2003 in accordance with WHO recommendations and national guidelines. The initial first-line regimen was d4T/3TC/EFV. To avoid d4T toxicity and because of EFV supply difficulties, the AZT/3TC/NVP combination was progressively introduced and has been the initial combination since July 2004.

Patients were clinically followed every month. CD4 counts were performed every six months. VL monitoring was not routinely available. Adherence support was provided by nurses through a programme of therapeutic patient education. After about 36 months of follow up, a cross-sectional clinico-immunological and virological study was performed in 2006: it revealed that 77% of the 309 included patients had shown virological success in an intent-to-treat analysis; if only ARV-naïve patients were considered, up to 83.5% had shown success [28].

Patients with first-line treatment failure in the ESTHER cohort were mostly detected through cross-sectional virological evaluations approved by the National Ethical Committee of Cambodia; these were performed in 2005 [29] and 2006 [28]. In addition, some patients failing their first-line ARV regimens were also routinely detected using clinico-immunological criteria. Patients in first-line treatment failure were eligible for a LPV/r-based second-line regimen.

### Study population

The present study is an analysis of the efficacy of LPV/r-based second-line regimens after 24 months of follow up, using routinely collected follow-up data.

As of 1 March 2009, all adults who had initiated a LPV/r-based second-line regimen more than 24 months earlier were eligible for the analysis. Medical background and follow-up information were routinely collected at each consultation on standardized forms and entered into an appropriate monitoring database.

### Immunological and virological follow-up assessments

Regular immunological (every six months) and virological assessments (once a year) were performed as part of the monitoring of adult patients who had been receiving HAART second-line regimens in the cohort. For the treatment success analysis after 24 months, the first viral load available after 24 months (VL24) of follow up on second-line HAART regimens was taken into consideration. HIV RNA viral loads (VL) was performed on -80°C frozen plasma at the Institut Pasteur du Cambodge, using the Agence Nationale de Recherche sur le Sida (ANRS) second generation (G2) real-time RT-PCR test [30,31]; this allows quantification of HIV-1 B and non-B subtypes, including the A/E subtype circulating in south-east Asia [32].

Using only 0.2 ml of plasma, the threshold of the assay was 250 copies/ml [31]. Genotypic resistance study was done in both the reverse transcriptase (RT) and the protease (PR) genes using available plasma specimen presenting detectable VL. Genotypic drug resistance interpretation was performed according to the latest version of the ANRS algorithm [33]. CD4 T cell counts were obtained using flow cytometry (Facscount, Beckton Dickinson and Cyflow, Partec, Germania).

### Statistical analysis

Patients who had not attended services for two or more months after their last scheduled appointments (i.e., three months of no visits as patients usually come to the clinic every month) and who could not be traced were classified as being lost to follow up and statistically considered on their last recorded visit to the clinics. CD4 gains compared with baseline at switch were calculated every six months after second-line HAART initiation. All analyses were performed using Stata 8.2 software (Stata Corp., Texas, USA).

## Results

Of those patients who had started a LPV/r-based second-line regimen, 70 who had started more than 24 months earlier were included in the analysis.

### Baseline characteristics and first-line HAART history

Baseline characteristics of the patients revealed that 51 of 70 (72.8%) were male and that median age was 40 years (interquartile range [IQR]: 37-46) (Table 1). The main initial first-line HAART regimens followed by these patients (Table 1) were AZT/3TC/NVP (33.3%), d4T/3TC/EFV (26.1%), d4T/3TC/NVP (17.1%) and AZT/3TC/EFV (18.8%). The median duration on first-line HAART regimens before switching to second-line regimens was 26.6 months (IQR: 15.2-29.4). At the time of the switch, most patients were already severely immunocompromised with a median CD4 count (n = 70) of 106 cells/mm^3 ^(IQR: 42-168). The median viral load at switch was 4.7 Log_10 _cp/ml (IQR: 3.1-5.4) (n = 65) (Table 1).

**Table 1:** Baseline characteristics of patients on second-line antiretroviral regimens for more than 24 months at the Calmette Hospital/ESTHER cohort in Phnom Penh, Cambodia

Characteristics	Second-line cohort (n = 70)
Male [n (%)]	51 (72.8)

Median age [years(IQR)*]	40 (37-46)

ARV* naïve before first line [n (%)]	15 (21.4)

PI naïve at switch [n (%)]	70 (100.0)

**Initial first-line ARV treatment**	

d4T/3TC/EFV [n (%)]	18 (26.1)

d4T/3TC/NVP [n (%)]	12 (17.1)

AZT/3TC/NVP [n (%)]	23 (33.3)

AZT/3TC/EFV [n (%)]	13 (18.8)

Other first lines [n (%)]	4 (5.8)

Follow-up time on first-line HAART* (months)	

Median (IQR)	26.6 (15.2-29.4)

**Characteristics at switch**	

CD4 T-cell counts (n)	70

Median nb cells/mm^3 ^[n (IQR)]	106 (42-168)

Count <50 cells/mm^3 ^[n (%)]	20 (28.6)

Median viral load (n = 65), [Log_10 _(IQR)]	4.7 (3.1-5.4)

**Prescribed second-line ARV treatment**	

ddI/3TC/LPV_/r _[n (%)]	46 (65.7)

TDF/3TC/LPV_/r _[n (%)]	5 (7.1)

TDF/ddI/LPV_/r _[n (%)]	7 (10.0)

AZT/ddI/LPV_/r _[n (%)]	6 (8.6)

AZT/3TC/LPV_/r_[n (%)]	2 (2.9)

Other second lines [n (%)]	4 (5.7)

**Follow-up time on second-line HAART (months)**	

Median (IQR)	37.3 (34.2-39.0)

* IQR: interquartile range, ARV: antiretroviral, HAART: highly active antiretroviral treatment

At switch, HIV drug resistance analysis in the reverse transcriptase (RT) gene was available for 41 out of 70 (58.6%) patients. Since these patients were supposed to be protease inhibitor (PI) naïve, HIV drug resistance analysis of the protease gene was not performed. The most common NRTI mutation at switch was the M184V mutation (n = 41, 100%). Among non-NRTI mutations, K103N (n = 20, 48.8%), G190A/S (n = 16, 39%) and Y181C/I (n = 9, 21.9%) mutations were most frequently observed.

All 41 patients displayed viruses resistant to both 3TC/FTC and NVP/EFV (n = 41, 100%). When looking at combinations of viral resistance among the 41 patients, 10 (24.4%) had viruses resistant to these four drugs only: 3TC/FTC and NVP/EFV; one (2.4%) displayed viruses also resistant to D4T. Twenty-five of them (60.9%) had viruses resistant to six drugs: 3TC/FTC, NVP/EFV and D4T/ZDV. Three patients (7.3%) displayed viruses resistant to seven drugs: 3TC/FTC, NVP/EFV, D4T/ZDV and ABC; and two patients (4.8%) showed resistance to eight drugs: 3TC/FTC, NVP/EFV, D4T/ZDV, ABC plus DDI.

Phylogenetic analysis of the RT gene [GenBank: HM026304 to GenBank: HM026346] revealed that 39 patients out of 41 (95.1%) were infected by CRF01_AE viruses and two (4.9%) by HIV-1 B-subtypes.

### Second-line ARV treatment

The most frequent second-line regimen used (Table 1) was ddI/3TC/LPV_/r _(n = 46, 65.7%). Based on the genotype results and the limited number of drugs available, some patients had to be switched to ddI/TDF/LPV_/r _(n = 7, 10.0%). The remaining patients were switched to TDF/3TC/LPV_/r _(n = 5, 7.1%), ddI/AZT/LPV_/r _(n = 6, 8.6%) or AZT/3TC/LPV_/r _(n = 2, 2.9%).

### Immunological outcomes on second-line ARV treatment

The median CD4 cell gain on second-line regimens (Table 2) was +80 cells/mm^3 ^(IQR: 30-152) at six months (n = 67), +134 cells/mm^3 ^[IQR: 71-204] at 12 months (n = 67) and +258 cells/mm^3 ^[IQR: 136-425] at 24 months (n = 63). Although these patients presented a very low baseline CD4 count at switch, only seven of 63 (11.1%, CI: 5.5- 2.1) patients had CD4 counts still below 200 cells/mm^3 ^at 24 months of second-line regimens.

**Table 2:** Immunological restoration of ESTHER patients on second-line regimens at Calmette Hospital in Phnom Penh, Cambodia

	At switch n = 70	M6 n = 67	M12 n = 67	M18 n = 63	M24 n = 63	M30 n = 46
Median CD4 count (cells/mm^3^)	106	197	258	308	372	344

(IQR)	(42-168)	(113-275)	(173-342)	(213-427)	(260-535)	(249-457)

% with CD4 count:						
<50 cells/mm^3^	28.6	1.5	3.0	3.2	0.0	0.0

<200 cells/mm^3^	54.3	50.7	31.3	20.6	11.1	15.2

Median CD4 count gain (cells/mm^3^)	-	+ 80	+ 134	+ 175	+ 258	+ 227

(IQR)	-	(30-152)	(71-204)	(99-279)	(136-425)	(134-403)

### Virological assessment of patients on second-line treatment

We analyzed the first VL available after 24 months of follow up (VL24) for all patients included in the study. At that time, three patients had died, two were lost to follow up, and 65 (92.8%, CI: 84.3- 96.8) were still on second-line treatment (for these, VL24 assessments were available). The median follow-up duration at the time of the VL24 evaluation was 27.4 months (IQR: 25.3-29.7). VL24 was undetectable (<250 cp/ml) for 60 of 65 patients (92.3%, CI: 83.2- 96.6) (Table 3). The VL24 of the five patients with virological failure ranged between 2.8 and 4.4 Log_10 _cp/ml (Table 4). Of these, three had received D4T/3TC/NVP, one had received D4T/3TC/EFV and one had received AZT/3TC/EFV as first-line regimens.

**Table 3:** Viral load measures of patients on second-line regimens for more than 24 months (n = 65)^a^

Viral load (copies/ml)	N	%	95% CI*	Cumulative%
<250	60	92.31	85.8 - 98.8	92.31

[250-1000]	1	1.54	0 - 4.5	93.85

[1000-10,000]	3	4.62	0 - 9.7	98.46

[10,000-30,000]	1	1.54	0 - 4.5	100.0

≥30,000	0	0.0		100.0

**Total**	65	100.0		100.0

^a ^taking into account the first viral load performed after 24 months of follow up (in practice ≥21 months of follow up) under second-line treatment. Lost-to-follow-up and dead patients (n = 5) excluded (follow up less than 24 months).

* CI: confidence interval

**Table 4:** Reverse transcriptase and protease resistance profiles of five patients with detectable viral load after 24 months on second-line antiretroviral regimens

Patient	Age	Gender	Second-line ARV regimens	Duration (months)	**VL24 Log**_**10**_	RT resistance profile	PR resistance profile
1	39	M	3TC DDI LPV/r	44	3.6	3TC FTC	not amplified

2	37	M	3TC DDI LPV/r	28	2.8	not amplified	not amplified

3	30	F	3TC DDI LPV/r	27	4.4	none	none

4	42	M	3TC TDF LPV/r	34	3.9	ABC TDF NVP EFV	IDV SQV/r* TPV/r*

5	48	M	LPV/r fosAPV	24	3.4	NPV EFV	IDV NFV ATV LPV/r TPV/r*

* Possible resistance

At VL24 evaluation (Table 4), RT and/or PR resistance profiles were available for four patients, all infected with CRF01_AE. HIV-PR genes of patients 1 and 2 (VL = 3.6 and 2.8 Log_10_, respectively) could not be amplified. RT and PR HIV drug resistance profiles of patient 3 (VL = 4.4 Log_10_) did not show any resistance-associated mutations. Patient 4 (VL = 3.9 Log_10_) showed resistance to the ABC, TDF, NVP and EFV reverse transcriptase inhibitors. This patient was also found to have HIV resistant to indinavir (IDV) and possibly to saquinavir (SQV) and tipranavir (TPV) [GenBank: HM026302]. Patient 5 (VL = 3.4 Log_10_) displayed HIV resistant to NVP, EFV, lopinavir (LPV), IDV, nelfinavir (NFV) and atazanavir (ATV). He also had viruses possibly resistant to TPV [GenBank: HM026303].

These five patients were boosted with second-line adherence counselling and followed up. Two of them died (patients 2 and 4) during this ongoing follow up (one died because of a traffic accident and one of renal failure). The other three were still followed on second-line HAART regimens as of 1 March 2009.

If we consider both dead and lost-to-follow-up patients as failure in an intention-to-treat analysis, our data reveal that 85.7% (60/70, CI: 75.6- 92.0) of the patients were showing second-line treatment success at the time of VL24 evaluation.

## Discussion

We report here on an analysis describing the outcomes of 70 HIV patients on LPV/r-based second-line HAART regimens for more than 24 months, followed at the ESTHER programme in Phnom Penh, Cambodia. After 24 months of follow up on second-line regimens, 65 (92.8%, CI: 84.3- 96.8) remained on treatment and 60 (92.3%, CI: 83.2- 96.6) had undetectable viral loads, giving an 85.7% (CI: 75.6- 92.0) treatment success rate in intent-to-treat analysis. A strong immune reconstitution was observed at 24 months (+258 cells/mm^3 ^[IQR: 136-425]) of follow up on second-line HAART regimens. While no specific information on adherence was available, these very good virological data revealed that these patients were indeed adhering well despite the complexity (number of pills) and the difficulties of storage conditions (at 2-8°C for LPV/r) of such regimens used at that time, demonstrating the feasibility of PI-based second-line regimens in such resource-limited settings.

Most of these patients were switched to second-line regimens following cross-sectional virological evaluations performed at the ESTHER/Calmette programme. Thus, it is important to note that this modality of switch is not representative of what is currently done in Cambodia, where clinico-immunological criteria are predominantly used. On the other hand, we could reasonably assume that such a cross-sectional survey might have detected treatment failures earlier than if routine immunological detection was applied.

Clearly, some patients at switch were already resistant to some second-line NRTI drugs recommended and available in Cambodia. Indeed, we found that all 41 patients with resistance genotype available before switching to LPV/r based second-line ARV regimens were resistant to 3TC/FTC, and that 10% (four of 41) were resistant to at least one other second-line drug, including ddI, ABC or TDF. Thus, we can assume that the vast majority of the 70 patients on PI-based regimens and followed up for 24 months were resistant to 3TC.

Because of national programme recommendations, a large majority of our patients (57%) initiated 3TC-based triple combinations as second-line HAART regimens. It is thus noteworthy to observe such excellent immuno-virological outcomes after two years of follow up despite the fact that such second-line regimens might have worked only as dual therapy or monotherapy from their initiation. This might be not surprising because all patients were apparently naïve for protease inhibitors.

On the other hand, the M184V mutation, in addition to conferring high levels of resistance to 3TC, has also been shown to reduce viral replication capacity [34], to increase the faithfulness of the RT [35], and to maintain more sensitivity to 3TC than previously believed [36,37]. This led to the speculation that 3TC might still be beneficial in patients harbouring the M184V mutation. Recent data from a pilot study comparing complete HAART interruption with the maintenance of 3TC despite the presence of M184V mutation in salvage therapy revealed better clinical and immunological outcomes at 48 weeks when maintaining 3TC, with no further accumulated mutations [38]. Whether such a benefit of 3TC also applies to patients on second-line regimens is still unknown and needs to be investigated.

In addition, it remains to be shown that the effectiveness of such "non-optimal" second-line regimens after two years could be maintained on longer periods of follow up. In any case, the fact that such patterns of resistance with limited options for second-line regimens was already observed following the initial cross-sectional survey advocates for the use of monitoring strategies allowing early virological failure detection, as well as the use of stronger first-line regimens with higher genetic barriers.

Considering the five patients presenting detectable VL after 24 months on LPV/r-based second-line regimens, we found that two of them (patients 4 and 5) harboured PI resistance-associated mutations. Further clinical investigations revealed that both of these two patients were exposed to several PIs and other RTIs before enrolment into the ESTHER cohort. Another patient (patient 3, Table 4), displayed no drug resistance mutation at all, which could be explained by treatment interruption. The PR gene could not be amplified for the two remaining patients (patients 1 and 2), probably because of their low-level viral loads (2.8 and 3.6 Log10). As for first-line ARV regimens, these data suggest that not being PI naïve could also be a risk factor for treatment failure on second-line regimens.

Recently, a multicohort study analyzing patients on second-line ARV therapy for more than six months in 27 Médecins Sans Frontières (MSF) ART programmes in Africa and Asia reported that 18.8% of 632 patients displayed WHO clinical, immunological or virological criteria of failure after a median of 11.9 months of follow up, with cumulative probabilities showing up to 28% failures at two years [20]. Our current study found only 7.7% (CI: 3.4- 16.8) virological failure among actively followed patients after 24 months, which is in apparent disagreement with the results reported by the MSF study. In fact, it is difficult to compare these studies since each used very different criteria to define second-line treatment failures.

Indeed, not all patients with clinical or immunological criteria of failure had virological confirmation in the MSF study, while all of our patients were defined virologically only. As it is known that patients with clinical or immunological WHO criteria of failure could display undetectable viral loads, it would be of interest to know the rates of viological failure among those patients with virological confirmation in the MSF study. In addition, the differences observed might be cohort specific since we analyzed a limited number of patients from one cohort only, while the MSF study analyzed 27 distinct cohorts and a much larger number of patients. Further studies addressing the effectiveness of boosted protease inhibitor-based regimens in resource-limited settings are still needed. Detailed description of cohort characteristics, including patient follow-up procedures and second-line adherence support, will be critical for identifying programmatic factors that might influence second-line outcomes in such settings.

## Conclusions

In conclusion, as reported by a recent Thai study [39], we have shown promising results concerning the feasibility and efficacy of PI/r-based second-line HAART regimens after two years of follow up in a cohort in Cambodia. However, further studies in Cambodia are needed to confirm such long-term favourable outcomes of second-line regimens in settings where first-line treatment failure detection and switching to second-line regimens do not use routine viral load and resistance genotyping, as in the ESTHER cohort.

These data also outline the need to work further on early treatment failure detection and on the affordability of additional second-line drug options to optimize second-line regimen choices. If current first-line regimens remain the same in resource-limited settings, randomized clinical trials will be critical to rapidly define the most appropriate second-line or even third-line regimens to be recommended. On the other hand, defining stronger, affordable, alternative first-line regimens in order to better preserve future second-line options might also be urgently warranted.

## Competing interests

The authors declare that they have no competing interests.

## Authors' contributions

EN, LF, CM and JFD conceived and designed the study protocol. VO, CH, OS and AD were in charge of the ESTHER patients' medical follow up. NL and IF contributed to the data collection in the field. JN and SN did the virological evaluation (viral load and resistance genotyping). LF did the statistical analysis. LF and EN led the writing of the manuscript; all investigators participated in its final writing and editing. All authors read and approved the final manuscript.
